# Development, inter-rater reliability and feasibility of a checklist to assess implementation (Ch-IMP) in systematic reviews: the case of provider-based prevention and treatment programs targeting children and youth

**DOI:** 10.1186/s12874-015-0037-7

**Published:** 2015-09-07

**Authors:** Margaret Cargo, Ivana Stankov, James Thomas, Michael Saini, Patricia Rogers, Evan Mayo-Wilson, Karin Hannes

**Affiliations:** 1 grid.1026.50000000089945086Spatial Epidemiology and Evaluation Research Group, School of Population Health, University of South Australia, Adelaide, Australia; 2 grid.83440.3b0000000121901201Evidence for Policy and Practice Information and Co-ordinating (EPPI) Centre, Social Science Research Unit, UCL Institute of Education, University College London, London, UK; 3 grid.17063.33Factor-Inwentash Faculty of Social Work, University of Toronto, Toronto, Canada; 4 grid.1017.70000000121633550Centre for Applied Social Research, RMIT University (Royal Melbourne Institute of Technology), Melbourne, Australia; 5 grid.21107.350000000121719311Johns Hopkins Bloomberg School of Public Health, Baltimore, MD USA; 6 grid.5596.f0000000106687884Methodology of Educational Sciences Research Group, Faculty of Psychology and Educational Sciences, KU Leuven, Leuven, Belgium

**Keywords:** Complex interventions, Children and youth, Provider-based interventions, Systematic reviews, Process evaluation, Implementation, Theory-driven reviews, Checklist development

## Abstract

**Background:**

Several papers report deficiencies in the reporting of information about the implementation of interventions in clinical trials. Information about implementation is also required in systematic reviews of complex interventions to facilitate the translation and uptake of evidence of provider-based prevention and treatment programs. To capture whether and how implementation is assessed within systematic effectiveness reviews, we developed a checklist for implementation (Ch-IMP) and piloted it in a cohort of reviews on provider-based prevention and treatment interventions for children and young people. This paper reports on the inter-rater reliability, feasibility and reasons for discrepant ratings.

**Methods:**

Checklist domains were informed by a framework for program theory; items within domains were generated from a literature review. The checklist was pilot-tested on a cohort of 27 effectiveness reviews targeting children and youth. Two raters independently extracted information on 47 items. Inter-rater reliability was evaluated using percentage agreement and unweighted kappa coefficients. Reasons for discrepant ratings were content analysed.

**Results:**

Kappa coefficients ranged from 0.37 to 1.00 and were not influenced by one-sided bias. Most kappa values were classified as excellent (n = 20) or good (n = 17) with a few items categorised as fair (n = 7) or poor (n = 1). Prevalence-adjusted kappa coefficients indicate good or excellent agreement for all but one item. Four areas contributed to scoring discrepancies: 1) clarity or sufficiency of information provided in the review; 2) information missed in the review; 3) issues encountered with the tool; and 4) issues encountered at the review level. Use of the tool demands time investment and it requires adjustment to improve its feasibility for wider use.

**Conclusions:**

The case of provider-based prevention and treatment interventions showed relevancy in developing and piloting the Ch-IMP as a useful tool for assessing the extent to which systematic reviews assess the quality of implementation. The checklist could be used by authors and editors to improve the quality of systematic reviews, and shows promise as a pedagogical tool to facilitate the extraction and reporting of implementation characteristics.

**Electronic supplementary material:**

The online version of this article (doi:10.1186/s12874-015-0037-7) contains supplementary material, which is available to authorized users.

## Background

The evaluation of complex interventions seeks to determine not only whether prevention and treatment interventions work but ‘what works in which circumstances and for whom?’ This phrase was originally coined by Pawson and Tilley [1] to reflect the logic of inquiry of the realist paradigm aimed at unpacking how interventions work to generate outcomes. Intervention mechanisms (i.e., how, why) and intervention modifiers (e.g., for whom, in which circumstances) are evaluated in the postpositivist paradigm using statistical mediation and moderation, respectively [2–4]. Despite between paradigm differences in the logic of inquiry there is a shared understanding on the need to provide context-relevant explanatory evidence beyond the main intervention effect. It is therefore crucial for evaluations to provide information on how programs were implemented and the factors influencing implementation. Understanding aspects of intervention implementation falls into the domain of process evaluation [5]. Process evaluation is an important component of an overall evaluation because it can help explain negative, modest and positive intervention effects, provide insight into the causal mechanisms of change including the conditions under which mediators are activated, and unpick those aspects of a multi-method/format (i.e., structured vs unstructured) intervention contributing to hypothesised intermediate and longer term outcomes [6–12]. At a minimum, it is recommended that process evaluations include information on reach, dose delivered, dose received, fidelity, recruitment and the contextual factors that influence implementation [12]. Contextual factors can be proximal (e.g., organizational resources, leadership) or distal to the program (e.g., geographic location). The inclusion of information on intermediate variables leading to hypothesised outcomes, formative/pre-testing procedures, and quality assurance measures is also recommended [12].

To situate implementation and the factors influencing implementation in relation to hypothesised change processes there has been an increasing reliance on the use of conceptual models, logic models or theory-driven approaches in evaluation [13] and systematic reviews [14].

Despite the importance of understanding ‘implementation in context’, intervention descriptions and process evaluation measures are poorly reported in medicine, social and psychological interventions [15–17], social work [18] and for a broad range of occupational health, public health and health promotion interventions [11, 19–21]. These deficiencies inhibit the translation and uptake of evidence by decision-makers with a mandate to improve specific outcomes or practices and additionally have spurred the development of reporting guidelines in primary studies. Current foci include the development of an extension of the Consolidated Standards of Reporting trials Statement for Social and Psychological Interventions (CONSORT-SPI) [22], the Oxford Implementation Index [23] and the Template for Intervention Description and Replications (TIDieR) [24]. Recently, a call was made for guidance on the process evaluation of complex public health interventions [10].

Although reviewers are constrained by reporting limitations in primary studies, guidance on the process evaluation of complex interventions would be informed by studies aimed at understanding the approaches, methods and tools used by reviewers to address aspects of intervention delivery and the factors influencing implementation. Such work may highlight exemplary practices and provide insight into the issues that need to be considered in guidance development.

Collaborators of the Campbell Collaboration Process and Implementation Methods Sub-Group (C2-PIMS) (http://www.campbellcollaboration.org/) undertook a review of systematic reviews to understand how reviewers approached implementation within their reviews. This study provides us with an opportunity to contribute to the development of methodological guidance on the process evaluation of complex interventions on this topic. The study casted an ‘implementation in context’ lens on understanding how complex interventions work to achieve their intended outcomes, in order to enable us to potentially detect Type III error or implementation failure, whether partial or complete, to be factored into explanations of intervention effectiveness [13].

We took a sample of reviews from the Campbell Collaboration Reviews of Interventions and Policy Evaluations (C2-RIPE) Library, focussing on provider-based prevention and treatment programs targeting children and youth. The choice for our sample was inspired by two intertwined arguments. Firstly, many prevention and treatment programs for children and young people can be usefully classified as complex–that is, they have several interacting components at multiple levels (e.g., school, home, community), which are tailored for different participants [25]. These programs may be delivered by diverse providers, such as teachers, psychologists, psychiatrists or health educators and often target changes in multiple behaviours (e.g., delinquent behaviour and smoking). Bringing about changes in these behaviours may require providers to utilise multiple strategies. Secondly government decision-makers and funding agencies are interested in this particular target group from a political point of view. Indeed, the antecedents of many behavioural problems, mental disorders, learning difficulties, and unhealthy lifestyle behaviours are established in childhood and adolescence. To prevent the development and ameliorate the effects of these problems federal, state, and local levels of government increasingly are calling for the use of evidence-based prevention and treatment interventions for children and families [26, 27]. Front-line staff and professionals are integral to the delivery of these programs [28, 29]. They play a key role in influencing children and youth’s knowledge, attitudes, beliefs and behaviours through direct interaction or by intervening on the environments (i.e., home, school) that shape children and youth’s development.

In the absence of a checklist to assess the degree to which process and implementation issues have been taken into account, a checklist for implementation (Ch-IMP) was developed. This is one of the first studies to tackle the issue of implementation at the systematic review level. This paper reports on the development of the checklist, its inter-rater reliability, reasons for discrepant ratings and feasibility of the checklist to assess ‘implementation in context’ for systematic reviews focusing on the delivery of provider-based prevention and treatment programs targeting children and youth. Implications for the future use of process evaluation checklists and guidance development are discussed.

## Methods

### Part one: checklist development and pretesting

#### Theoretical framework

The Ch-IMP captured whether implementation measures and processes were assessed within provider-based child and youth prevention and treatment reviews and, if so, which measures and processes were addressed and how they were addressed. Reviews may not consider implementation at all or may pinpoint one or more dimensions which may be reported qualitatively, descriptively or in the meta-analyses. The checklist was designed to identify a broad range of dimensions within these programs and assess how included dimensions were integrated within reviews.

Chen’s conceptual framework [30] for program theory was selected as the framework to inform the development of the Ch-IMP for multiple reasons. First, other models feature implementation but they tend to focus on a specific aspect of implementation (i.e., fidelity) [31–34]. Chen’s framework, on the other hand, is comprehensive and features process evaluation and the contextual factors influencing implementation. The framework also features providers as central to program delivery which corresponds with the study focus on the implementation of provider-based prevention and treatment interventions targeting children and youth. The framework is supported by open systems theory which recognises that context shapes implementation and program outcomes. This fits with the notion of complex interventions as applied in health, medicine, education, social work, criminal justice and psychology.

**Fig. 1:**
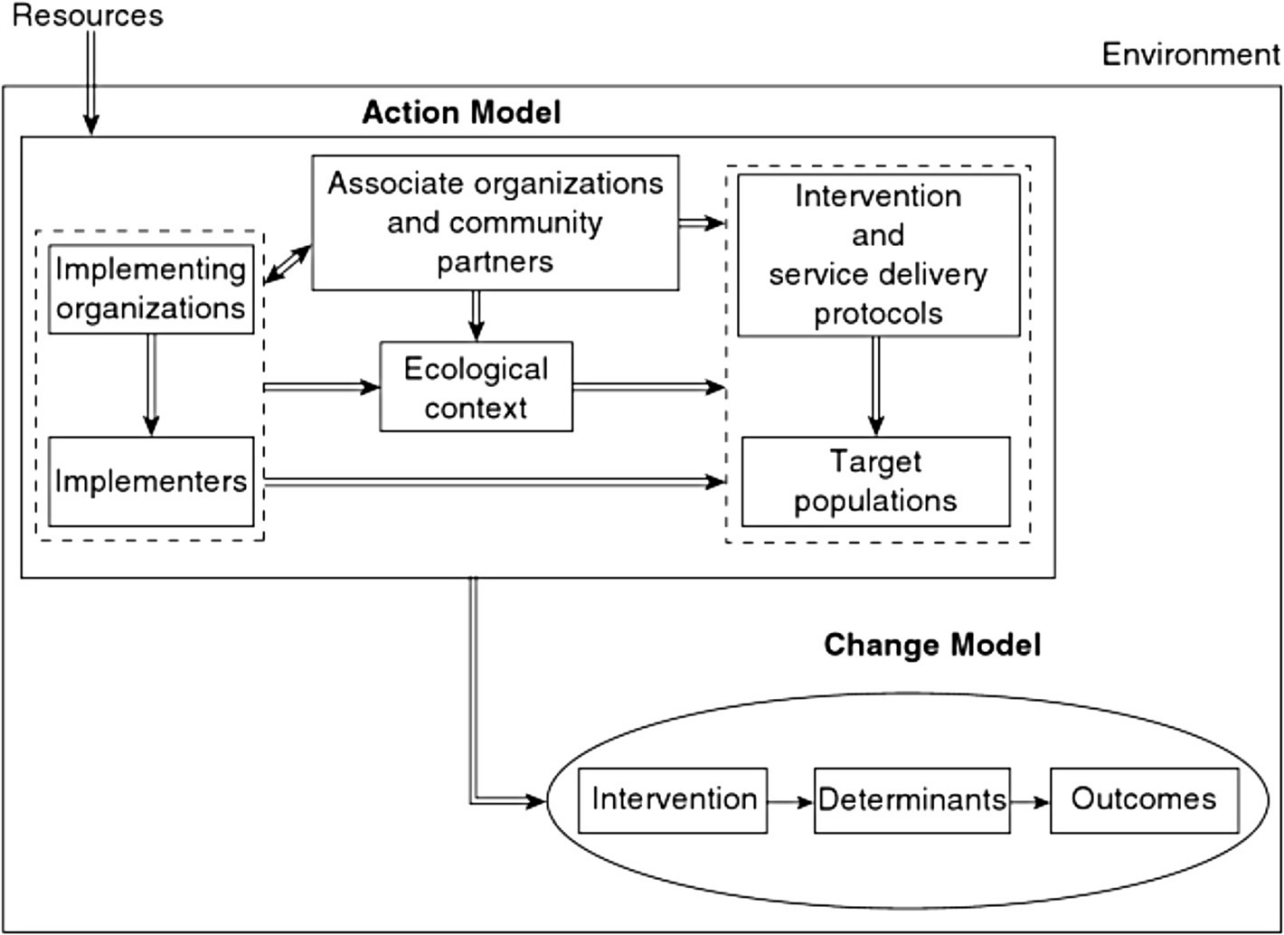
Conceptual framework for developing program theory. Source: Chen H-T. Practical Program Evaluation. Thousand Oaks, CA: Sage Publications, 2005. Reprinted with permission from Sage Publications

In Chen’s framework (Fig. 1) the action model supporting the prevention or treatment intervention must be implemented appropriately in order to activate the transformation process in the program’s change model. The action model articulates what the program will do to bring about change in children and youth outcomes. For example, if a change model for a given intervention is designed to increase children’s levels of physical activity by changing perceived social norms for physical activity and opportunities to engage in physical activity, the action model stipulates what the intervention will do to activate the change model. Will the intervention include school-based activities only? Will parents be engaged? Will teachers receive training? Will the school collaborate with external agencies? Which agencies, how and why? Who will the intervention target and why? The action model provides the justification for these choices and clarifies what the program will do (i.e., program operations) to increase behaviour change related to physical activity.

**Fig. 2:**
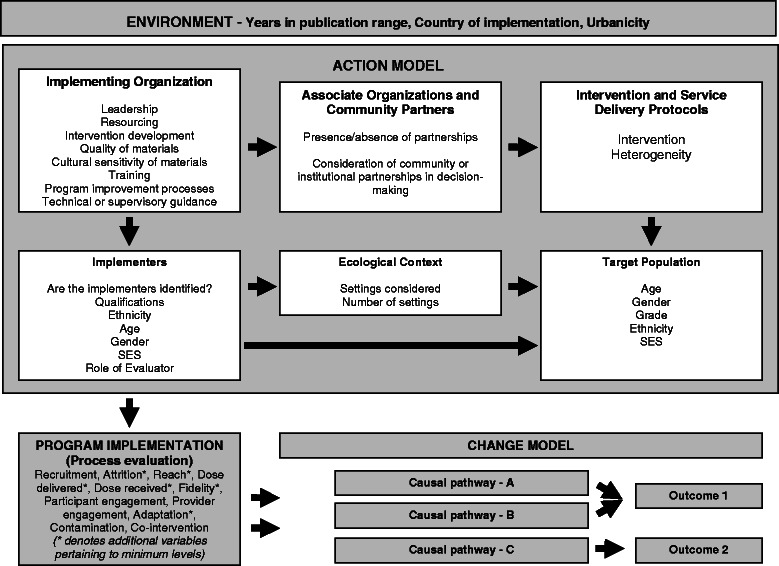
Items in the Checklist for Implementation (Ch-IMP) that correspond with Chen's framework for program theory

*Action model*: The action model is the program plan that supports the delivery of provider-based child and youth prevention interventions. It considers the day-to-day planning for arranging staff, resources, settings and support organizations so the intervention delivers its services to targeted children and youth. For the purposes of the Ch-IMP, the action model is comprised of the child or youth prevention or treatment intervention, target population (i.e., children, youth or their caregivers), implementers (i.e., teachers, mentors, community volunteers, professionals), implementing organization (i.e., school, community organization), associate organizations and community partners (i.e., between a school and a non-profit agency) and ecological context (i.e., intervention strategies may implicate delivery in school, community or clinical settings).

The prevention or treatment intervention is supported by (1) an intervention protocol that outlines the orientation, structure and content of the intervention and the nature of children, youth or parents’ exposure to the content and (2) a service delivery protocol that operationalises the steps that need to be taken to implement the intervention in the field [30]. Intervention heterogeneity can be assessed in relation to core strategies, elements, activities, components or types; these will vary according to the specific intervention.

For the studies included in our review in which the Ch-IMP was tested, children and youth are the target population or ultimate target group designated to benefit from the program. Some programs, however, act on more than one level and involve more than one type of participant (e.g., parents, siblings, and target children) [35]. This was one of the factors contributing to a more complex level of the review.

Providers (implementers) are integral to program delivery and are recruited for their qualifications (e.g., clinical psychologists, speech therapists) or relevant previous experiences or interest (e.g., volunteers, mentors). Providers may attempt to deliver interventions as they were originally designed but can encounter obstacles to delivery according to pre-specified criteria. For some types of programs (i.e., child and youth mentoring programs), pre-existing provider characteristics such as age, gender and ethnicity are important considerations. For example, cultural identification plays a role in the engagement of young people from minority or Indigenous populations [36].

The implementing organization is the lead organization responsible for providing resources to deliver the program. For child and youth prevention and treatment programs, the implementing organization may be a school, non-profit organization or community organization, for example. These organizations may provide a range of supports such as training their staff (e.g., teachers, counsellors, and volunteers), technical assistance, feedback mechanisms and monitoring.

Partnerships may be established between implementing organizations and associate organizations and community partners that have a stake or interest in the intervention.

If linkage with these entities is not properly established it could compromise access to the target population (e.g., referrals from local police to community mentoring organization) or volunteers (e.g., mentoring organization with educational institutions). Specialised programs may be externally developed and implemented either by external providers or staff in the implementing organization who are trained by the external agency (i.e., pregnancy prevention program implemented in schools by an external agency). Partnerships with associate organizations and the community can lead to interaction effects including collaborative advantage and the achievement of outcomes that neither organization could have achieved on their own [37]. Where associations with external organizations are not identified, assumptions may be made about the role of the implementing organization in program delivery.

The ecological context reflects the broader social systems within which children, youth and their caregivers receive or engage with the intervention. Interventions may be implemented in one or more settings (e.g., school, community, organization, home). Operating procedures, the formality of procedures, organizational norms and power structures may vary across settings and influence program delivery and the responsiveness of the target population to the intervention.

*Program implementation*: Chen’s framework suggests that the intervention effect is a *joint effect* of implementing the intervention and implementing the factors in the action model.

The first component of this joint effect pertains to *implementing the intervention* which is captured through *process evaluation* measures. For provider-based prevention and treatment interventions targeting children and youth, these measures include exposure of intended intervention components to the treatment and control participants (i.e., contamination, co-intervention, program differentiation); implementers delivering the required number of sessions and strategies to participants (i.e., dose delivered); participants use, consumption or interaction with the intervention components (i.e., dose received); participants actual participation in the program (i.e., reach); participant drop-out rates (i.e., attrition), participant’s attitudes or feeling about the program (i.e., participant engagement) and provider’s attitudes or feelings about the program (i.e., provider engagement). Information provided in the intervention and service delivery protocols specifies the nature of intended program delivery, which is the essence of many definitions of fidelity. These definitions can range from ‘faithful replication,’ to ‘the degree to which specified procedures are implemented as planned’ [38]. Some definitions of fidelity, however, are broader and include adherence, exposure or dose, quality of delivery, participant responsiveness and program differentiation [31].

The second component of the joint effect pertains *to implementing the factors in the action model*. These factors–implementing organization, implementer, associate organizations and community partners, target population, ecological setting–are defined in the preceding section. From a programmatic perspective, information on the factors influencing implementation can be found in the intervention and service delivery protocols, which include the conceptual model, program theory or logic model underpinning the program. For example, a program manual may specify that the implementing organization is required to offer a specific type of training for implementers to deliver a multi-component clinical intervention to children.

*Change model*: The change model specifies how implementation of the intervention and elements of the action model bring about the primary outcome through a set of intermediate impacts. The change model can be articulated in an a priori program theory or conceptual model that outlines how the program will activate the change pathways; it can also be depicted graphically in a logic model [13]. Specifying the mediators in the causal pathway(s) is key to the change model. The change model can have a single or multiple outcome pathways depicting one or more mediators depending on the characteristics of the intervention, participant and other factors at play in the implementing system [39].

*Environment*: Contextual factors external to the action model can shape how programs are planned (positively, negatively or neutral), implemented and received. Usually, programs are initiated through resources acquired from the external environment which lead to the development of an action model. These may include aspects of the geographic environment, historical period, political environment and include broader social norms, for example. Contextual factors may be identified in the theoretical assumptions of the intervention protocol and potentially the risk assessment of a project management plan.

#### Checklist item selection

In line with the broader program theory orientation of Chen’s framework [30], the checklist assessed aspects of the action model, program implementation, change model and environment.

Table 1 provides the definitions for each item in the action model, program implementation, change model and environment (Fig. 2). Stem questions are framed as one of the following: ‘Was the [item] considered?’, ‘Has the review considered aspects of [item]?’, ‘Does the review consider [item]?’ Given the study focus on implementation, items are concentrated in the former two domains. Questions were derived from documents retrieved from a review of the published and grey literatures. More specifically, published articles were obtained from an information retrieval project that examined how process evaluation and theory-driven evaluations were indexed in Medline, Embase, Psychinfo and Eric electronic databases. This led to the retrieval of a large pool of articles which provided the basis for the development of the checklist. In addition, websites of systematic review organizations were reviewed. The worldwide web was searched for grey literature. Authors’ broad-based experiences conducting evaluations and systematic reviews of provider-based prevention and treatment programs for children and youth facilitated the identification of grey literature and seminal works in theory-driven evaluation/reviews, process evaluation, and contextually sensitive provider-based interventions. The following documents from the relevant content areas and agencies informed the development of the checklist: implementation/process evaluation literature [8, 12, 31, 38, 40–43] theory-driven evaluation literature [13, 30, 34, 44, 45] systematic review literature [46, 47] reporting guidelines and recommendations [48–52], the Cochrane Collaboration [53, 54], the EPPI-Centre [55], the Society for Prevention Research [56] the U.S. Centres for Disease Control and Prevention Community Guide [57].

**Table 1:** Domains and items within each domain for the Checklist for Implementation (Ch-IMP)

Action model		
Intervention & service delivery protocol	Intervention heterogeneity	Whether consideration was given to the range of strategies, elements, activities, types of components for the interventions included within the review. If relevant, please use the comment function to elaborate on the specific aspects of intervention heterogeneity captured in the review.
Target population	Age	Specific age or age range of participants
	Gender	Gender of participants
	Grade	Specific grade or grade range of participants
	Ethnicity	Ethnic background of participants
	Socio-economic status	Income, highest level of education, occupation of caregivers of participants
Implementers	Implementer identified^a^	Identify who implements the intervention and interfaces with participants.
	Qualifications	Consideration to different types of implementers; please consider whether reviews considered implementer’s education level, certifications, or past relevant experiences to assess their ability to do the job.
	Ethnicity	Ethnic background of the implementers.
	Age	Specific age or age range of implementers.
	Gender	Gender of implementers.
	SES	One or more of implementer’s income, highest level of education or occupation.
	Role of evaluator	Whether role of the evaluator was addressed. (i.e., roles in program delivery vs evaluation).
Implementing organization	Leadership	Whether program champions and leaders provide instructions or guidance to staff/ implementers to facilitate the intervention delivery.
	Resourcing	Resources includes having sufficient personnel/ staff, financial resources/ operational budget, space, buildings or sites (physical resources), and materials/ equipment (technological resources) to run the program.
	Intervention development	Intervention development can be strengthened through strategic program planning and program design processes including intervention mapping, needs assessment, pilot-testing, formative evaluation, evaluability assessment or other developmental work.
	Quality of materials	The quality of materials is commonly assessed in relation to the quality of the intervention materials (e.g., activity materials, curriculum) or the training materials/manual.
	Cultural sensitivity	Interventions that consider the language, socio-cultural values and traditions may be considered more appropriate to the cultural groups in which they are intended to benefit.
	Training	Assess whether any consideration has been given to training, the quality of training or any other aspect of training that acts to enhance the skills/ competency of service delivery staff.
	Program improvement processes	Information from intervention improvement processes such as performance monitoring, feedback, formative evaluation, intervention monitoring can improve delivery.
	Technical or supervisory guidance	Providing implementers with practical or expert support and guidance (unrelated to intervention improvement processes) during their implementation efforts to improve implementation quality.
Associate organizations & community partners	Presence/absence of partnership	Note any formal partnerships or collaborations during intervention planning or implementation
	Other partnership processes	Note one or more aspects of the collaboration or partnership such as pooling resources, dividing responsibilities for different aspects of complex intervention delivery.
Ecological context	Settings considered	Please specify whether this review formally considered the setting in which the intervention was implemented.
	Settings^b^	Please specify whether the number of settings in which the interventions were implemented.
Program implementation (process evalution)		
Recruitment		Refers to specific information on the procedures used to recruit participants into or attract participants to the intervention.
Attrition		Attrition is a measure of drop-out rates, or the proportion of participants lost during the course of an intervention or during follow up.
Minimum attrition		Please determine whether the review considered a minimum attrition/drop-out rate.
Reach		Reach refers to the degree to which the intended audi ence participates in an intervention by ‘their presence’.
Minimum reach		Please determine whether the review considered implementation of minimum reach.
Dose delivered		Dose delivered is established through the efforts and actions of implementers or implementing organization. This concept refers to the proportion or amount of an intervention (or the combined strategies) delivered to participants; often measured through frequency (e.g., twice per week), duration (e.g., duration of program in months) and intensity (e.g., total ^a^ program delivery hours).
Minimum dose delivered		Please determine whether the review considered implementation of minimum dose delivered.
Dose received		Dose received, also referred to as exposure, is a characteristic of the target populations’ engagement and active participation in an intervention. It is an objective measure of the extent to which participants actually utilise and interact with program strategies, materials, or resources.
Minimum dose received		Please determine whether the review considered implementation of minimum dose received.
Fidelity		Intervention fidelity is a commonly used measure in process evaluation. It has been conceptualised and measured in a variety of ways. Its essential definition reflects the extent to which an intervention is implemented as originally intended by program developers. It has been applied to assessing intervention strategies to the integrity of an implementing system (i.e., “the extent to which an intervention has been implemented as intended by those responsible for its development”; “closeness between the program-as-planned and the program-as-delivered”; “faithful replication”; the degree to which “specified procedures are implemented as planned”). Please use the comment function to provide the definition used in the review.
Minimum fidelity		Please determine whether the review considered implementation of minimum fidelity.
Adaptation		The extent to which program content is intentionally or purposefully changed during implementation, from the original standard, to enhance program effectiveness. Programs can be adapted to be situationally responsive to local needs and circumstances. Please note the reasons for adaptation.
Minimum adaptation		Please determine whether the review considered implementation of minimum adaptation.
Participant engagement		Refers to the subjective attributes that define their participation in, interaction with or receptivity to an intervention. This can include what they think of the program (cognitive orientation) such as satisfaction with the program, commitment, perceived relevance of the program of the outcomes or how they feel about the program (affective orientation) such as enthusiasm or enjoyment.
Provider engagement		Implementer engagement refers to the subjective attributes of program staff that can influence their capacity to deliver intervention strategies. This can include: a) what staff think about the program content (cognitive orientation) such as satisfaction with the program, commitment, motivation, perceived importance/ buy-in, perceived relevance of the program of the outcomes; b) how staff feel when implementing the program (affective orientation) such as enthusiasm or enjoyment; or c) staff’s interpersonal style or the methods used to communicate concepts (e.g., warmth, empathy).
Co-intervention		When interventions other than the treatment under study are applied differently to the treatment and control/comparison groups.
Contamination		When an intervention is unintentionally delivered to participants in the control group or inadvertent failure to deliver the intervention to the experimental group.
Change model		
A priori change model^c^		The Change Model links intervention strategies to a sequence of short, intermediate and longer-term observable and intended outcomes. This sequencing of outcomes is referred to as an *outcome chain*. It specifies what needs to change within people or their environments (short to intermediate term impacts) for the longer-term intervention outcome to be achieved. The Change Model explains how and why the change in participants happens. Assess whether the review provides a description of how the interventions work to achieve outcomes with consideration to the activities and strategies that are intended to bring about change. Some reviews will include multiple Change Models.
Logic diagram used^c^		Please specify whether the review provides a graphical depiction of how each intervention works to achieve its short, intermediate and long-term outcomes. These diagrams are also referred to as ‘logic models’ or ‘theory of change’ diagrams. The sequence of outcomes (short term to long-term) should be linked to intervention strategies or activities.
Environment (external context)		
Years		Years in which primary studies were published. Can be used as a proxy measure for historical/period effects.
Country		Name of county of program delivery. May act as a proxy for political climate, availability of resources, social norms.
Regions or areas within countries		Areas and regions within countries may be specified and may include remoteness or urbanicity indices (e.g., rural, remote/metropolitan, northern/southern). May act as a proxy for access to resources or some other measure.

^a^Stem Question: Are the implementers clearly identified–dichotomous Yes or No response scale

^b^7 category nominal response scale: 1, 2, 3, 4, 5+, Not specified, Unclear

^c^4 category nominal response scale: No, Yes (articulation clear), Yes (articulation unclear), Other

A draft version of the checklist was trialled by three raters on three provider-based prevention and treatment reviews (two raters completed all three reviews and one rater completed one review within this set), and iteratively revised through discussion, and input from members of the C2-PIMS. Raters provided open-ended comments for each question to capture the adequacy of the response scale, and identify any issues with the definitions or wording of questions.

### Part two: piloting the checklist

#### Study selection

Reviews were selected from the Campbell Collaboration Library if they met the following inclusion criteria: a) published by March 2010; b) included at least one study; and c) reported outcomes separately for children or youth aged 0–22 years. Of the 58 published C2 reviews, 27 reviews met the inclusion criteria following screening by two authors (Additional file 1).

#### Data collection instrument

The pilot version of the Ch-IMP comprised 47 items and captured; 1) variables pertaining to elements of the action model (n = 25); 2) whether reviews articulated change models supported by a broader intervention model or theory (n = 2); 3) variables pertaining to elements of the process evaluation (n = 17); 4) dimensions of the environment (n = 3); 5) variables unique to each review; 6) open-ended comments for each question (from study raters); and 7) issues, challenges and implications for the key domains as articulated by review authors and identified by study raters.

Of the 47 questions, 43 questions utilised a 7 category nominal scale (Table 2), one question utilised an alternate 7 category nominal scale, two questions utilised a 4 category nominal scale, and one question utilised a dichotomous yes/no scale. The latter three variations in the scale are noted in the footnote of Table 1. The 7 category response scale was designed to identify variables ‘not considered’ in the review, those that reviewers intended to extract information about but could not due to reporting limitations in primary studies (i.e., ‘intended but unable’) and variables which reviewers intended to extract but did not report on within the review (i.e., ‘intended but not reported’). The scale was also designed to identify gaps in reporting in primary studies and to provide some indication on whether implementation was formally considered within reviews. Any occurrence of a variable was considered as present, but if there was only one mention in an in-text narrative summary or summary table in the appendix of the review, the variable was reported at the descriptive ‘quantitative unsynthesised’ level. If information on a variable was present and synthesised across primary studies in the review, it was coded as descriptive ‘quantitative synthesised’ whilst information linked to meta-analysis was coded as ‘linked to meta-analysis’. Information on variables that did not fit into these categories was coded as ‘other’ and a comment was provided to justify this selection.

**Table 2:** Seven-category response scale used for 45 of 47 items in the checklist for implementation (Ch-IMP)

a	No, not considered	The dimension is not formally considered in the review.
b	No, intended but unable	No, the review intended to address the dimension but was unable to on the basis of limited information provided in primary studies.
c	No, intended but not reported	No, the review intended to report on the measure of interest but no information is provided in the analysis or discussion section.
d	Yes, quantitative unsynthesised	Yes, descriptive information is provided on the dimension for one or more studies (e.g., in a narrative summary or table in an appendix) but the information is not synthesised across studies.
e	Yes, quantitative synthesised	Yes, descriptive quantitative information is synthesised across studies (e.g., percentage or range provided in a table or narratively).
f	Yes, linked to meta-analysis	Yes, the dimension is linked to meta-analysis; effect measures calculated.
g	Other	Information on the dimension is provided that is unclear, ambiguous or does not fit the above categories. The measure of interest has been considered but is homogenous. Please comment on why this response category was selected.

#### Data extraction

Two researchers independently reviewed and extracted the information from the 27 reviews. Information was extracted on hard copy and uploaded into EPPI-Reviewer [58]. An instruction guide was developed concurrently to support application of the checklist and used to guide the extraction process. The items in the Ch-IMP that correspond with domains in Chen's framework are shown in Fig. 2.

For example, the rater would read the review and search for information pertaining to the target variable “intervention development” defined as the following in the code book:

**Table:** 

Was any consideration given to intervention development?
• Intervention development can be strengthened through strategic program planning and program design processes [13] including intervention mapping, concept mapping, needs assessment, pilot-testing, formative evaluation, evaluability assessment or other developmental work. This does not include adaptation of the intervention–either purposely or non-purposivefully (when reasons for adaptation are not provided). Should this be encountered, please refer to the Adaptation question under Process and Implementation.

The rater would check one box in the checklist (below) and add open ended comments to the question in the open space in the box.
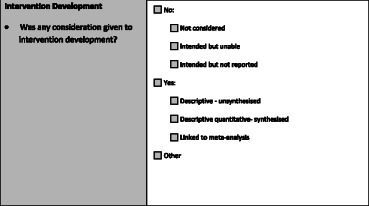


In the case of the target variable ‘fidelity’ the reviewer would examine the systematic review for information corresponding to the following definition provided in the code book, and follow the same process.

**Table:** 

Was fidelity assessed, that is, the degree to which interventions are implemented as intended by its developers?
Intervention fidelity is a commonly used measure in process evaluation. It has been conceptualised and measured in a variety of ways. Its essential definition reflects the extent to which an intervention is implemented as originally intended by program developers. It has been applied to assessing intervention strategies to the integrity of an implementing system (i.e., “the extent to which an intervention has been implemented as intended by those responsible for its development”; “closeness between the program-as-planned and the program-as-delivered”; “faithful replication”; the degree to which “specified procedures are implemented as planned”). Please use the comment function to provide the definition used in the review.


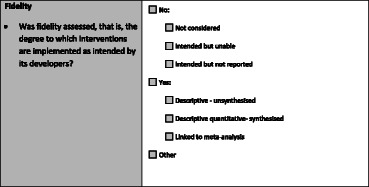


The information was uploaded to EPPI-Reviewer when the domain was complete or when the review was complete. Given the length of the checklist and the fact that the information in the reviews did not always appear in the order of the checklist, the raters found it helpful to complete the ratings on hard copy prior to uploading the information. A screen shot of EPPI-Reviewer below illustrates the fidelity measure with a link to highlighted text in the review. Comments could be added for the selected response using the info box.
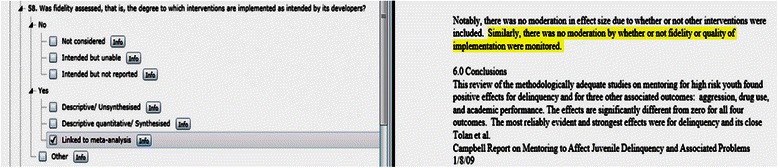


#### Data analysis

Inter-rater reliability analysis assessed consistency among raters for each of the 47 items. The unweighted kappa statistic was used to correspond with the 7 category nominal response scale. Reporting the kappa statistic alone is appropriate when the marginal totals in the tables are relatively balanced. However, when the prevalence of a given response is either very high or low, the kappa value may indicate a low level of reliability even though the observed proportion of agreement is high [59, 60]. Because paradoxical values of kappa may occur due to a skewed distribution, we report the percentage agreement between raters and AC1 statistic. The latter is considered a more robust measure of agreement on the basis that it is less influenced by differences in response category prevalence [60]. Following Cichetti and Sparrow [61], kappa values were rated as fair (0.40–0.59), good (0.60–0.74) or excellent (0.75–1.0). Kappa values below 0.40 indicate poor agreement. Analyses were conducted using WinPEPI. Reasons underlying discrepant ratings were documented and content analysed for categories and sub-categories using NVivo qualitative software.

## Results and discussion

### Reliability

**Fig. 3:**
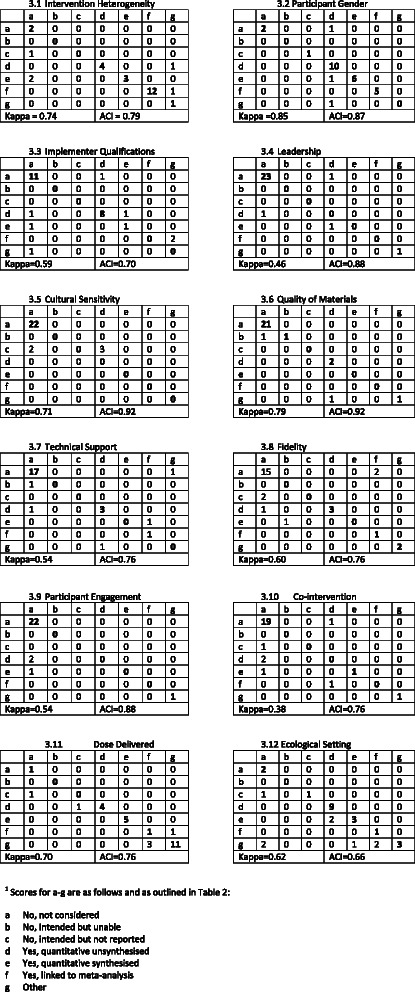
Rater (n = 2) scores for 12 measures in the checklist for implementation (Ch-IMP)

Table 3 displays results for percentage agreement between the two raters, kappa coefficients with 95 % confidence intervals, and AC1 coefficients for the 47 items in the Ch-IMP. Twelve tables are shown in Fig. 3 to illustrate the nature of disagreements between raters.

**Table 3:** Inter-rater reliability results for 47 items in the checklist for implementation (Ch-IMP) (n = 27 reviews)

	Percentage agreement	Kappa (95 % CI)	AC1 statistic (95 % CI)
	Action model		
Intervention and service delivery protocols			
Intervention Heterogeneity	82	0.74 (0.56–0.93)	0.79 (0.62–0.96)
Target population			
Age	85	0.80 (0.63–0.98)	0.83 (0.68–0.98)
Gender	89	0.85 (0.68–1.00)	0.87 (0.74–1.00)
Grade	85	0.75 (0.57–0.92)	0.84 (0.69–0.99)
Ethnicity	82	0.74 (0.54–0.94)	0.79 (0.62–0.96)
SES	74	0.62 (0.41–0.84)	0.71 (0.52–0.90)
Implementers			
Implementer identified	100	1.00	
Qualifications	74	0.59 (0.37–0.82)	0.70 (0.50–0.89)
Ethnicity	96	0.84 (0.53–1.00)	0.96 (0.89–1.00)
Age	96	0.82 (0.53–1.00)	0.96 (0.89–1.00)
Gender	96	0.78 (0.37–1.00)	0.96 (0.89–1.00)
Socio-economic status	100	1.00	
Role of the evaluator	96	0.90 (0.70–1.00)	0.96 (0.88–1.00)
Implementing organization			
Leadership	89	0.46 (0.03–0.89)	0.88 (0.76–1.00)
Resourcing	100	1.00	
Intervention development	93	0.72 (0.34–1.00)	0.92 (0.82–1.00)
Quality of materials	93	0.79 (0.53–1.00)	0.92 (0.82–1.00)
Cultural sensitivity	93	0.71 (0.34–1.00)	0.92 (0.82–1.00)
Training	82	0.64 (0.40–0.88)	0.80 (0.63–0.96)
Program improvement processes	93	0.68 (0.33–1.00)	0.92 (0.82–1.00)
Technical or supervisory guidance	78	0.54 (0.26–0.81)	0.76 (0.58–0.93)
Associate organizations and community partners			
Presence/absence of partnership	93	0.81 (0.4 8–1.00)	0.92 (0.81–1.00)
Other partnership proc	96	0.79 (0.45–1.00)	0.96 (0.89–1.00)
Ecological context			
Settings considered	74	0.62 (0.42–0.82)	0.66 (0.46–0.86)
# Settings	48	0.37 (0.15–0.59)	0.40 (0.18–0.62)
Process evaluation			
Recruitment	82	0.68 (0.46–0.91)	0.80 (0.63–0.96)
Attrition	74	0.63 (0.41–0.85)	0.71 (0.52–0.90)
Minimum attrition	74	0.47 (0.20–0.74)	0.72 (0.53–0.90)
Reach	93	0.82 (0.59–1.00)	0.92 (0.81–1.00)
Minimum reach	96	0.65 (0.02–1.00)	0.96 (0.89–1.00)
Dose delivered	79	0.70 (0.50–0.90)	0.76 (0.58–0.93)
Minimum dose delivered	85	0.45 (0.03–0.86)	0.85 (0.70–0.99)
Dose received	89	0.54 (0.10–0.98)	0.88 (0.76–1.00)
Minimum dose received	100	1.00	
Fidelity	78	0.60 (0.33–0.87)	0.76 (0.58–0.93)
Minimum fidelity	100	1.00	
Adaptation	82	0.67 (0.42–0.92)	0.80 (0.63–0.96)
Minimum adaptation	100	1.00	
Participant engagement	89	0.54 (0.10–0.98)	0.88 (0.76–1.00)
Provider engagement	93	0.64 (0.17–1.00)	0.92 (0.82–1.00)
Co-intervention	78	0.38 (0.03–0.73)	0.76 (0.59–0.94)
Contamination	74	0.42 (0.12–0.71)	0.72 (0.54–0.90)
Change model			
Apriori intervention model	78	0.65 (0.40–0.89)	0.72 (0.52–0.92)
Logic diagram used	100	1.00	
Environmment			
Years	74	0.63 (0.41–0.86)	0.71 (0.52–0.89)
Country	74	0.65 (0.45–0.85)	0.70 (0.51–0.90)
Urbanicity	78	0.67 (0.45–0.90)	0.75 (0.57–0.93)

Inter-rater agreement ranged from 48 to 100 % Kappa coefficients ranged from 0.37 to 1.00. The majority of kappa values were classified as excellent (n = 18) or good (n = 17) with fewer items falling into the fair (n = 6) or poor (n = 2) categories. The prevalence-adjusted coefficients, deemed more robust to the influence of response category prevalence, indicates good or excellent agreement for all items in which percentage agreement was high and the kappa coefficient was low.

Our inter-rater reliability results indicate paradoxical values of kappa as reflected in the high percentage agreement rates and low corresponding kappa coefficients. A limitation of the kappa statistic is that it is affected by category prevalence. For example, the percentage agreement for the ‘leadership’ target variable in the implementing organization was high (89 %) with 23 agreements rated as “not considered” yet the kappa coefficient was fair with a very wide confidence interval (κ = 0.46, 0.03–0.89). In this instance and many others, as evident in Table 3, the kappa over-estimates chance agreement, thus reducing the estimated kappa value. If this paradox is present, an interpretation based exclusively on the kappa value may be misleading. Although there is no consensus on which specific statistics to report, there is consensus that statistics adjusting for prevalence must be reported in conjunction with kappa values [60, 62].

The wide confidence intervals for the kappa coefficients are of concern. At the time we conducted this study, 27 reviews met the inclusion criteria. It is recommended that sample sizes should not consist of less than 30 comparisons as the standard error is sensitive to sample size. Although confidence intervals can be calculated for studies with small sample sizes, these are likely to be wide, resulting in kappa values of 0.40 or less, which indicate poor or no agreement. Examination of the confidence intervals for the kappa coefficients shows that 18 items indicate poor agreement or no agreement. The AC1 coefficients, on the other hand, are above 0.40 in all confidence intervals except for the ecological settings variable.

The 7 category response scale is a variation and elaboration of the three-level categorisation of yes/done, no/not done or can’t tell/unclear often used in checklists [63–65]. Because the goal of this study was to inform the development of guidance by understanding how the target variables in the action model, change model, environment and implementation were represented in the reviews, we elaborated on the ‘no’ and ‘yes’ response categories, specifically to gain this insight. Elaboration of this scale is shown in Table 2.

Although the unweighted kappa coefficients are wide-ranging and some are influenced by prevalence bias, the majority of prevalence-adjusted coefficients are in the acceptable range. In light of the lack of consensus on which prevalence adjusted statistic to use, we are cautiously optimistic about the pilot findings on inter-rater reliability and look to the qualitative data to provide insight into the basis for disagreements. These results should be interpreted in the context of the single data source of Campbell Collaboration reviews that was utilised.

### Sources of disagreement

As shown in Table 4, and highlighted below, the reasons for scoring discrepancies were related to: 1) information missed during the extraction; 2) issues with clarity or sufficiency of information provided in the review; 3) issues encountered with the tool; and 4) issues encountered at the level of the review.

**Table 4:** Reasons for inter-rater disagreement

Information missed in extraction	
a.	Missed information in text
b.	Missed information in in-text or summary tables in appendices
c.	Missed information in a multi-dimensional measure
Information on target variables and processes being unclear	
a.	Lack of justification provided for target variables
b.	In some instances it was unclear whether a target variable was defined on the basis of information present or absent in primary studies
c.	Information provided in the review made it difficult to assign the target variable to a response category
d.	In the absence of intervention theories or models linking intervention strategies to process, impacts and outcomes, it was difficult to interpret some variables
Limitations of the tool	
a.	Definitions in the tool did not adequately capture the heterogeneity in the target variable
b.	Target variable has a two-part question which can lead to inconsistent ratings
c.	Response category definitions
d.	Definitions of the term is too narrow
e.	Multiple indicators for a single measure
f.	Reviews with one primary study
Limitations of the review	
a.	Inconsistency in the presentation of target variable in the review
b.	Location of information in the review not in expected sections
c.	Lack of sub-headings
d.	Tables of summary characteristics

One rater missing an occurrence of a target variable was a strong contributor to discrepant scoring. This was attributed to use of a 7 category response scale (Table 2) which included the response category ‘quantitative unsynthesised’ to capture any occurrence of a target variable in the in-text narrative summary or summary table in the appendix of the review. This issue is illustrated in Fig. 3.2–3.4 and 3.7–3.10. For example, one review of narrative summaries for 44 primary studies spanned 40 pages [66] and one rater missed information reported on the target variable, ‘leadership.’ This contributed to one of the coding discrepancies observed in Fig. 3.4. It should be noted that the frequency of endorsement for categories 2 and 3 was very low. As shown in Fig. 3.1, 3.5–3.8, 3.10 and 3.12 these variables also were coded as ‘not considered’ by one rater which contributed to discrepant ratings.

The lack of clarity and definition in target variables and processes proved problematic. In some instances it was unclear whether a target variable was defined on the basis of information present or absent in primary studies. The statement, “*As these programs were relatively simple, none of the evaluators reported problems with implementation of the program*” [67] led one rater to query whether this assessment was based on the consistent reporting of fidelity in primary studies or because none of the studies reported problems. Coding discrepancies in participant engagement and provider engagement emerged in multi-level studies, such as parenting programs in which parents received a provider-based program and outcomes were assessed on both parents and children [68]. The target population was interpreted differently and led to ratings of ‘not considered’ and ‘quantitative synthesised’ by the two raters (Fig. 3.9). We additionally found that process evaluation measures were operationalized differently in reviews. For example, depending on the program, dose delivered can be operationalised as the number of educational sessions delivered per week, program duration [69, 70] or in a school feeding program, it can reflect the percentage recommended daily allowance for energy [71]. Conversely, for non-standardised interventions such as multi-systemic therapy, where clients are referred to treatments based on their initial assessment [35], dose delivered varied according to participant and family exposure to specific intervention components. The presence of intervention models in these reviews may have facilitated interpretation of outcome and process evaluation measures.

As might be expected with a pilot study, some issues were encountered with the tool. Definitions for ecological setting captured differences in broad setting types (i.e., home, school) but did not adequately capture within-setting variation. For example, some school-based reviews looked at how outcomes for children varied according to special classes or regular classes. Furthermore, our definitions for co-intervention, contamination and fidelity could have been more inclusive to capture the diversity of terms used across the reviews. Some reviews referred to implementation problems [69, 70] which were coded as fidelity (linked to meta-analysis) by one rater and not considered by the second rater, as illustrated in Fig. 3.8. Discrepant ratings for contamination and co-intervention were influenced by the use of different terms, for example program differentiation [72] and performance bias [72–74]. This led to differences in coding of ‘not considered’ for one rater and quantitative synthesised for the second rater (Fig. 3.10). The literature indicates that these terms can span aspects of co-intervention and contamination. We additionally found that many reviews used multiple dose delivered measures which were coded as ‘other’ by both raters (Fig. 3.11). The checklist is not good for assessing multiple measures of the same target variable; these measures may be expressed differently within the review (i.e., one measure may be quantitative synthesised and a second measure linked to meta-analysis). We also found that the checklist does not work well for reviews comprising only one study due to limited heterogeneity [1] and subsequent differential ratings of ‘not considered’ or ‘other’ for some variables.

Finally, review level issues contributed to scoring discrepancies. In some instances, the specification of variables changed within the review. For example, a parenting review initially targeted females and males (as parents) but excluded fathers as stated in the discussion section [68]. Discrepancies also arose due to the location of information in the review. In some cases, information on target variables, like country, appeared for the first time within the discussion instead of the methods or results sections. The presentation and/or organization of information in reviews contributed to scoring discrepancies by making it easier for a rater to miss information. This can be seen in Fig. 3.1, 3.2 and 3.12 where one rater has scored ‘quantitative synthesised’ or ‘linked to meta-analysis’ and the other rater has selected a different category (i.e., not considered, quantitative unsynthesised or quantitative synthesised). To this end, it would have been helpful for reviews to use subheadings such as ‘process evaluation’ to clearly identify relevant variables of interest [71]. Furthermore, consistent extraction of information from primary studies, within summary tables, could greatly have improved clarity relating to the availability of information relating to target variables. Clear identification of these variables can provide insight into what information reviewers are looking for and thus whether the absence of such information is due to its omission in primary studies or if it represents a reporting issue at the review-level.

The reasons for discrepant scoring underscore the need for two reviewers to extract information from primary studies. This is particularly important at the pilot stage of tool development so the reasons underpinning the discrepancies can be used to improve the tool and the review process.

### Feasibility

The Ch-IMP was developed as part of a larger project aimed at understanding how systematic reviewers address implementation in effectiveness reviews. This was seen as an important starting point for developing guidance to assist reviewers in addressing implementation. This resulted in the use of a 7 category response scale to detect variations in implementation assessment and the factors influencing implementation. As shown in Table 2, response categories 2 (i.e., intended to assess but unable) and 3 (i.e., intended to assess but not reported in primary study) were designed to identify reporting issues in primary studies and at the review-level. Category 4 was designed to pick up any occurrence of a target variable in the review. In addition, the checklist was designed to capture open-ended comments on each question and domain, and identified review-specific measures. These comments and measures are not reported in this study, but contributed to the overall response burden of the tool. The majority of reviews required 4–6 h to complete using the checklist. The length of time was influenced by the level of detail (i.e., narrative summaries, number of tables and appendices), the organization/presentation of material (i.e. headings, sub-headings, definition of and consistent use of terms) and the media utilized for data entry. For the latter, both paper and software (i.e., EPPI-Reviewer) were used. The initial Ch-IMP contained 85 variables. The response burden led to the extraction of a core set of 47 variables from the full set. Questions not included in this report pertain to whether variables listed in Table 2 were considered in the search strategy, inclusion/exclusion criteria, sensitivity analysis and risk of bias.

The feasibility of the checklist would be improved by streamlining the response scale from a 7 category to a 3 category (yes, no, other) response scale, improving the definitions corresponding to the domains and elements of Chen’s framework, improving the instruction guide and having raters enter extracted information in only one platform (i.e., paper or software).

## Conclusions

Pilot results suggest that the 47 item Ch-IMP is a promising checklist for assessing the extent to which systematic reviews of provider-based prevention and treatment programs targeting children and youth have considered the impact of implementation variables. We hope that further evaluations using the checklist may draw attention to the importance of addressing implementation in effectiveness reviews and that it may be used by reviewers to facilitate the systematic extraction and reporting of these measures and processes. To our knowledge this is the first theoretically-informed implementation checklist for complex interventions. Chen’s conceptual framework for program theory [30] was the basis for the development and application of the checklist. The application of the checklist was tested on a narrow sub-set of complex interventions, specifically provider-based prevention and treatment programs geared for children and youth as the target population. We argue that all of the domains are important to the checklist (i.e., action model, change model, implementation and external environment) but that elements within some domains (i.e., implementers) and specific measures (i.e., provider engagement and target population grade) will need to be dropped or adapted for the review of non-provider (i.e., exclusively technology or self-help interventions) or policy-based interventions (i.e., taxation) and interventions that are not focused on children or youth as the target population Because the checklist was developed as part of a broader project aimed at gaining insight into the assessment of implementation in systematic reviews, a 7 category response scale was used to detect variations in implementation assessment and reporting. This response scale, however, may be less useful for reviewers interested in examining whether certain process evaluation measures have been considered using a 3-level nominal scale (i.e., yes, no, other) and may need to be adapted for wide-scale use.

Our experiences from this pilot suggest the following implications for the future use of checklists or development of guidance to strengthen the assessment or reporting of implementation in reviews. First, reviewer’s use of different terms to refer to the same process evaluation target variable suggests a need for a comprehensive glossary with clear examples that illustrate the application of terms. For example, the glossary needs to recognise that some process evaluation measures such as dose delivered and dose received will have program-specific operationalisations. Such a glossary should also be sensitive with regards to the reasons for differing terminology; for example, some interventions will be tailored intentionally for specific situations, and the language of “implementation fidelity” would be inappropriate when describing such practice. Second, given inconsistencies in the reporting of implementation in reviews and the use of different definitions, we recommend that two raters extract information from primary studies. Third, we recommend a checklist with fewer than seven response categories, particularly if there is little interest in pinpointing the gaps between what reviewers intended to assess in their reviews and what they were not able to report due to reporting limitations in primary studies. The time required to complete such a fine-grained assessment can compromise reliability by contributing to rater response burden. Fourth, a priori intervention models with clearly defined and situated variables may help reduce the uncertainty in interpreting process evaluation measures and the factors influencing implementation in reviews. Finally, the presentation and layout of reviews such as the use of subheadings (i.e. process evaluation, how interventions might work), the definition of terms, and consistent reporting of information in summary tables, may reduce discrepant ratings and differential interpretation of results. This would also make it easier to pinpoint the nature of reporting limitations in primary studies by making transparent the discrepancies between what reviews intended to measure and what information was not available in primary studies. This paper is the first in a set of papers that will be used to support the development of guidance to assess implementation in effectiveness reviews; subsequent papers will report on pilot results from application of the Ch-IMP, elaborate on program implementation findings and adaptation of the tool for primary studies. Future adaptations of the tool will be informed by usability testing to improve the efficiency of the tool.

## Additional file

Additional file 1:
**Campbell Collaboration systematic reviews included in the review of reviews.** (PDF 211 kb)

## Abbreviations

C2: Campbell Collaboration
C2-PIMS: Campbell Collaboration Process and Implementation Methods Subgroup
C2-RIPE: Campbell Collaboration Reviews of Intervention and Policy Effectiveness
Ch-IMP: Checklist for Implementation
