# Supplementary material for: Development, inter-rater reliability and feasibility of a checklist to assess implementation (Ch-IMP) in systematic reviews: the case of provider-based prevention and treatment programs targeting children and youth
Source: BMC Med Res Methodol. 2015 Sep 7;15:73. doi: 10.1186/s12874-015-0037-7 (PMC4562191; doi:10.1186/s12874-015-0037-7)
Supplement: Additional file 1: — Campbell Collaboration systematic reviews included in the review of reviews. (PDF 211 kb) [file 12874_2015_37_MOESM1_ESM.pdf]

Additional file 1: Campbell Collaboration systematic reviews included in the review of reviews

	Authors	Year	Title
1	Armelius, Bengt-Ake & Andreassen, Tore H	2007	Cognitive-Behavioral treatment for antisocial behavior in youth in residential treatment
2	Barlow, Jane & Parsons, Jacci	2005	Group-based parent-training programmes for improving emotional and behavioral adjustment in 0-3 year old children
3	Coren, Esther & Barlow, Jane	2004	Individual and group based parenting for improving psychosocial outcomes for teenage parents and their children
4	Ekeland, Eilin ; Abbott, Joanne ; Hagen, Kare B; Heian, Frode & Nordheim, Lena	2005	Exercise to improve self-esteem in children and young people
5	Farrington, David & Ttofi, Maria	2009	School-Based Programs to Reduce Bullying and Victimization
6	Garrido, Vicente & Morales, Luz Anyela	2007	Serious (violent and chronic) juvenile offenders: A systematic review of treatment effectiveness in secure corrections
7	Kristjansson, Elizabeth ; Farmer, Anna P; Greenhalgh, Trish ; Janzen, Laura ; Krasevec, Julia ; MacDonald, Barbara ; MacGowan, Jessie ; Mayhew, Alain ; Petticrew, Mark ; Robinson, Vivian ; Shea, Beverley J; Tugwell, Peter & Wells, George A	2007	School feeding for improving the physical and psychosocial health of disadvantaged students
8	Law, James ; Garrett, Zoe & Nye,Chad	2005	Speech and language therapy interventions for children with primary speech and language delay or disorder
9	Littell, Julia ; Forsythe, Burnee ; Popa, Melanie & Austin, Sabrina	2005	Multisystemic therapy for social, emotional, and behavioral problems in youth aged 10-17
10	Macdonald, Geraldine ; Higgins, Julian & Ramchandani, Paul	2006	Cognitive-behavioral interventions for children who have been sexually abused
11	Macdonald, Geraldine & Turner, William	2008	Treatment Foster Care for improving outcomes in children and young people
12	Mayo-Wilson, Evan ; Dennis, Jane & Montgomery, Paul	2008	Personal assistance for children and adolescents (0-18) with intellectual impairments
13	Mishna, Faye ; Cook, Charlene ; MacFadden, Robert ; Saini, Michael & Wu, Meng-Jia	2009	Prevention and intervention of cyber abuse targeting children and adolescents: A systematic review to evaluate current approaches
14	Nye, Chad; Schwartz, Jamie & Turner, Herbert	2006	Approaches to parent involvement for improving the academic performance of elementary school age children
15	Petrosino, Anthony ; Buehler, John &	2007	Scared Straight and other juvenile awareness programs for preventing juvenile deliquency

	Turpin-Petrosino, Carolyn		
16	Petrosino, Anthony ; Turpin-Petrosino, Carolyn & Guckenburg, Sarah	2009	Formal System Processing of Juveniles: Effects on Delinquency
17	Piquero, Alex R; Farrington, David ; Jennings, Wesley G; Tremblay, Richard & Welsh, Brandon	2008	Effects of early family/parent training programs on antisocial behavior and delinquency
18	Piquero, Alex R; Jennings, Wesley G & Farrington, David	2010	Self-control interventions for children under age 10 for improving self-control and delinquency and problem behaviors
19	Ritter, Gary ; Albin, Ginger ; Barnett, Joshua ; Blankenship, Virginia & Denny, George	2006	The effectiveness of volunteer tutoring programs: A systematic review
20	Scher, Lauren ; Maynard, Rebecca A & Stagner, Matthew	2006	Interventions intended to reduce pregnancy-related outcomes among adolescents
21	Tolan, Patrick ; Bass, Arin ; Henry, David & Schoeny, Michael	2008	Mentoring interventions to affect juvenile delinquency and associated problems
22	Turner, William ; Dennis, Jane & Macdonald, Geraldine	2007	Behavioral and cognitive behavioral training interventions for assisting foster carers in the management of difficult behavior
23	Wilson, Sandra Jo & Lipsey, Mark W	2008	The effects of school-based social information processing interventions on aggressive behavior: Part II: Selected/indicated pull-out programs
24	Wilson, Sandra Jo & Lipsey, Mark W	2006	The effects of school-based social information processing interventions on aggressive behavior: Part I: Universal programs
25	Winokur, Marc ; Holtan, Amy & Valentine, Deborah	2009	Kinship Care for the Safety, Permanency, and Well-Being of Children Removed from the Home for Maltreatment
26	Zief, Susan G; Lauver, Sheri & Maynard, Rebecca A	2006	Impacts of after-school programs on student outcomes
27	Zwi, Karen ; O`Brien, Tracey ; Tait, Paul ; Wheeler, Danielle ; Williams, Katrina & Woolfenden, Sue	2007	School-based education programmes for the prevention of child sexual abuse
